# Neuroprotective Effect of Schisandra Chinensis on Methyl-4-Phenyl-1,2,3,6-Tetrahydropyridine-Induced Parkinsonian Syndrome in C57BL/6 Mice

**DOI:** 10.3390/nu11071671

**Published:** 2019-07-21

**Authors:** Chi-Lin Li, Yang-Hwei Tsuang, Tung-Hu Tsai

**Affiliations:** 1 Institute of Traditional Medicine, School of Medicine, National Yang-Ming University, Taipei 112, Taiwan; 2 Department of Orthopedic Surgery, Taipei Medical University-Shuang Ho Hospital, Ministry of Health and Welfare, New Taipei City 23561, Taiwan; 3 Department of Orthopedic Surgery, Taipei Medical University Hospital, Taipei Medical University, Taipei 106, Taiwan; 4 Graduate Institute of Acupuncture Science, China Medical University, Taichung 40402, Taiwan; 5 Department of Education and Research, Taipei City Hospital, Taipei 103, Taiwan; 6 Department of Chemical Engineering, National United University, Miaoli 36063, Taiwan

**Keywords:** *Schisandra chinensis* extract, botanical medicine, Parkinson’s disease, MPTP, dopamine, oxidative stress

## Abstract

*Schisandra chinensis* (Turcz.) Baill. (*S. chinensis*) is a well-known botanical medicine and nutritional supplement that has been shown to have potential effects on neurodegeneration. To investigate the potential neuroprotective effect of *S. chinensis* fruit extract, 1-methyl-4-phenyl-1,2,3,6-tetrahydropyridine (MPTP) was used to induce behavioral disorders and dopaminergic neuronal damage in mice, and biochemical indicators were examined. Male C57BL/6 mice were used to establish the MPTP-induced parkinsonian syndrome model. Open field and rotarod tests were performed to evaluate the overall manifestation of motor deficits and rodent motor coordination. The mice were divided into 8 groups as follows: normal control; MPTP alone (25 mg/kg, i.p.); *S. chinensis* extract pretreatment (0.5, 1.5, 5 g/kg, p.o.); and *S. chinensis* extract treatment (0.5, 1.5, 5 g/kg, p.o.). Liquid chromatography coupled to electrochemical detection was used to monitor neurochemicals in the striatum. Tyrosine hydroxylase content was measured by immunohistochemistry, and biochemical antioxidative indicators were used to evaluate the potential neuroprotective effects of *S. chinensis* fruit extract. The results demonstrated that treatment with *S. chinensis* fruit extract ameliorated MPTP-induced deficits in behavior, exercise balance, dopamine level, dopaminergic neurons, and tyrosine hydroxylase-positive cells in the striatum of mice. Among the pretreated and treatment groups, a high dose of *S. chinensis* fruit extract was the most effective treatment. In conclusion, *S. chinensis* fruit extract is a potential herbal drug candidate for the amelioration and prevention of Parkinson’s disease.

## 1. Introduction

Parkinson′s disease (PD) is a common progressive central nervous system degenerative disease that commonly occurs in people over 50 years of age [1]. The onset of PD is mainly due to the death of dopaminergic neurons in the substantia nigra pars compacta (SNpc), leading to the loss of dopamine in the striatum. The primary symptom of PD is motor impairment, including bradykinesia, tremor, rigidity, postural instability and hypokinesia [2]. Even though the underlying pathogenesis of PD remains unknown, inflammation, mitochondrial dysfunction and oxidative stress may play a pivotal role in the neurodegeneration associated with PD [3,4]. Research has demonstrated that oxidative stress and inflammation cause dopaminergic neurons to undergo apoptosis, leading to PD [5].

It is well known that 1-methyl-4-phenyl-1,2,3,6-tetrahydropyridine (MPTP) is a neurotoxin that causes a Parkinson-like syndrome in primates [6], humans [7] and mice [8]. MPTP rapidly passes through the blood-brain barrier (BBB) because it is a highly fat-soluble compound [9]. When MPTP enters the brain, it is rapidly metabolized through monoamine oxidase type B (MAO-B) into 1-methyl-4-phenylpyridinium (MPP^+^) [10], which is taken up by dopaminergic neurons and accumulates in the mitochondria [11]. Then, MPP^+^ is selectively absorbed by the dopaminergic neurons through the dopamine transporter and blocks mitochondrial respiration via inhibiting complex I, thus inducing an increase in reactive oxygen species (ROS) and a reduction in ATP [12]. Recently, an ex vivo chronic (96 h) model was developed that reproduced the features of the human pathology [13].

The botanic medicine, *S. chinensis* was first described in a book entitled *Compendium of Materia Medica* (in Chinese: Pen-Tsao-Kang-Mu) in 1817 by Li Shih-Chen. In western-based medicine and traditional Chinese medicine, *S. chinensis* is a very popular natural product with medicinal value that is widely used to treat anxiety and insomnia, enhance physical strength and recovery, and as a sedative [14] or tonic [15]. *S. chinensis* is commonly known as Chinese magnolia-vine in the family Schisandraceae, and more than 40 Schisandra lignans have been isolated. Previous studies have highlighted its many biological activities, such as anticarcinogenic [16], hepatoprotective [17], anti-human immunodeficiency virus [18,19], antioxidant [20] and anti-inflammatory [21] effects. Furthermore, Schisandra lignans have revealed protective neuronal effects against neurotoxicity induced by glutamate, 6-hydroxydopamine, and amyloid-β and lipopolysaccharide [22,23,24] and showed conservatory effects against neuronal cell damage in conditions such as stroke, Alzheimer′s disease and other neurodegenerative diseases.

Based on the above literature review, we hypothesized that the botanical extract of *S. chinensis* possesses several neuro-activities, which may indicate potential efficacy in the prevention and improvement of PD. To investigate our hypothesis, the MPTP-induced parkinsonian syndrome model was applied. The herbal extract was administered before and after the disease model was induced by MPTP to represent the prevention and amelioration of the experimental disease status, respectively. The aim of this study was to investigate the effects of *S. chinensis* on MPTP-induced behavioral disorders and dopaminergic neuron damage in C57BL/6 mice. The experimental design was as follows: (1) animal behaviors were evaluated with open field and rotarod tests; (2) the levels of dopamine (DA), homovanillic acid (HVA) and 3-methoxy-4-hydroxyphenylacetic acid (DOPAC) in the striatum were monitored; (3) ROS levels, including the levels of glutathione peroxidase (GPx), catalase (CAT) and superoxide dismutase (SOD) in the blood, were evaluated; and (4) the number of tyrosine hydroxylase (TH)-positive cells in the striatum was determined using immunohistochemistry staining.

## 2. Materials and Methods

### 2.1. Chemicals and Reagents

Dopamine, sodium 1-octanesulfonate monohydrate, 5-HT, HVA, 5-HIAA, sodium metabisulfite and DOPAC were obtained from Sigma-Aldrich Chemicals (St. Louis, MO, USA). Sodium chloride, hydrochloric acid, sodium dihydrogen phosphate, ethylenediaminetetraacetic acid disodium, sodium hydroxide, and acetonitrile were purchased from E. Merck (Darmstadt, Germany). MPTP hydrochloride was purchased from T.C.I. Co., LTD. (Tokyo, Japan)

### 2.2. Herbal Preparation of Aqueous Ethanol Extract from S. Chinensis

The fruit parts of *S. chinensis* (product lot no. Q0606) were purchased from Chia-Hui Inc., Taipei, Taiwan. The extraction of *S. chinensis* was performed according to the guidelines of the Ministry of Health and Welfare, Taipei, Taiwan, as follows: (1) the dry fruits of *S. chinensis* (1.2 kg) were decocted with ethanol-water (1:1 v/v) (16 L) in a stewpot for approximately 4 h at 80 °C; (2) alcohol was removed from the extract solution using decompression concentrator; and (3) water was removed by freeze-drying. Then, the *S. chinensis* extract was stored in a refrigerator. The *S. chinensis* weighed 330 g, and it was stored for additional quantification by LC–MS/MS.

### 2.3. Experimental Animals

**Figure 1:**
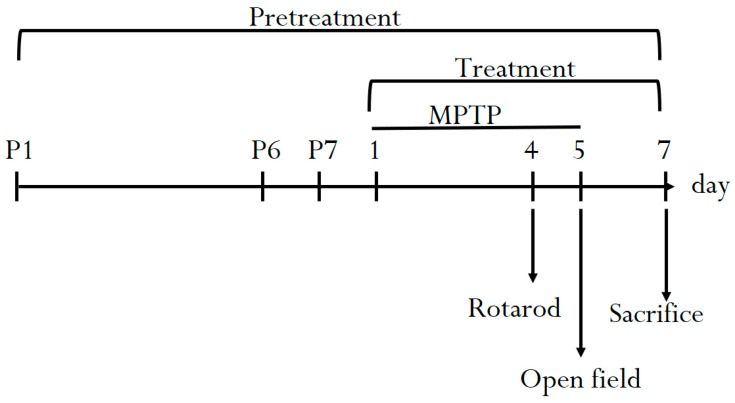
Schematic diagram of the experimental procedure. The pretreated group was administered *S. chinensis* (0.5, 1.5 and 5 g/kg, p.o.) for seven consecutive days before and MPTP administration (25 mg/kg, i.p.). The treated group was administered with *S. chinensis* (0.5, 1.5 and 5 g/kg, p.o.) for seven consecutive days of MPTP administration (25 mg/kg, i.p.). The behavioral experiments were performed on the 4th day after MPTP administration (25 mg/kg, i.p.). Pretreatment indicates that oral *S. chinensis* was administered for 7 days (p1–p7); then, MPTP was administered for five consecutive days, and *S. chinensis* was administered for 7 consecutive days. Treatment indicates that MPTP was administered for 5 consecutive days; then, *S. chinensis* was administered for 7 consecutive days. MPTP indicates that MPTP was administered for 5 consecutive days. MPTP, 1-methyl-4-phenyl-1,2,3,6-tetrahydropyridine.

All animal experiments were reviewed and approved by the National Yang-Ming University Laboratory Animal Care and use committee. Sixty-four C57BL/6 mice were purchased from the experimental animal center of National Yang-Ming University, Taipei, Taiwan. The animals could freely obtain food (laboratory rodent diet 5P14, PMI Feeds, Richmond, IN, USA) and water. The dose of *S. chinensis* for mice was derived from a human dose by following a conversion equation recommended by the U.S. Food and Drug Administration guidelines as follows: human equivalent dose (mg/kg) = animal dose (mg/kg) × (animal km/human km) [25]. The km factor, which is the body weight (kg) divided by the body surface area (m^2^), is used to convert the mg/kg dose in the study to the mg/m^2^ dose. The km factors are 3 and 37 for mice and humans, respectively. All 64 animals were stochastic separated into eight groups. Each group comprised 8 mice and were treated as follows: Group I, normal control, received triple-deionized water (1 mL/kg); Group II MPTP (25 mg/kg, i.p.); Group III, MPTP treatment (25 mg/kg, i.p.) + *S. chinensis* pretreatment (0.5 g/kg, p.o.); Group IV, MPTP treatment (25 mg/kg, i.p.) + *S. chinensis* pretreatment (1.5 g/kg, p.o.); Group V, MPTP treatment (25 mg/kg, i.p.) + *S. chinensis* pretreatment (5 g/kg, p.o.); Group VI, MPTP treatment (25 mg/kg, i.p.) + *S. chinensis* treatment (0.5 g/kg, p.o.); Group VII, MPTP treatment (25 mg/kg, i.p.) + *S. chinensis* treatment (1.5 g/kg, p.o.); Group VIII, MPTP treatment (25 mg/kg, i.p.) + *S. chinensis* treatment (5 g/kg, p.o.). The experiment lasted 14 days. The mice were pretreated (groups III–V) with the various doses of *S. chinensis* extract before MPTP administration, and then MPTP was administered for five days. MPTP (25 mg/kg, i.p.) was administered intraperitoneally once per day for five subsequent days. During MPTP administration, respective extract treatment for each group was performed 1 h prior to MPTP administration. The behavioral study included the open field test and the rotarod test, which were executed 5 days after MPTP administration. On the final day, the mice were sacrificed, and the striata were separated for further neurotransmitter analysis and immunohistochemistry studies (Figure 1).

### 2.4. Apparatuses, Analytical Conditions and Sample Preparation for Neurochemicals

The high-performance liquid chromatography with electrochemical detection (HPLC-ECD) system consisted of a Decade II electrochemical detector fitted with a SenCell electrochemical flow cell (Antec, Zoeterwoude, The Netherlands), a CMA240 sample injector with a 20-μL loop (CMA Microdialysis AB, Kista, Sweden), a CMA200 refrigerated microsampler, and a BASi PM-92E LC pump (Bioanalytical Systems, West Lafayette, IN, USA). An Inertsil ODS-3 column (250 × 4.6 mm, i.d., 5 μm, GL Sciences, Tokyo, Japan) was maintained at ambient temperatures. The optimized mobile phase was composed of a mixture of a water phase and acetonitrile at a ratio of 86:14 v/v. The water phase was composed of 100 mM sodium dihydrogen phosphate buffer, 100 mM citric acid, 600 mg/L OSA and 0.1 mM Na_2_EDTA with the pH adjusted to 3.0 using a 10 M sodium hydroxide solution. The flow rate was set at 1.0 mL/min, and the injection volume was 15 μL. The analytes were detected at a range of 2 nA, a filter value of 0.05 Hz, and a detection potential of 0.65 V versus the reference electrode. Data processing was performed using Clarity software system (DataApex, Prague, Czech Republic).

On the final day after drug administration, the animals were sacrificed. The brains were removed immediately, and the striatum was dissected out on ice and stored at −80 °C until use. For HPLC analysis, the samples were homogenized in 10 volumes of ice-cold stock solution (0.1 M HClO_4_, 0.1 mM EDTA, 0.1 mM Na_2_S_2_O_5_ in deionized water). The homogenates were then centrifuged at 16,000× *g* at 4 °C for 10 min and filtered in 0.22-μm filter membrane. Finally, an aliquot (15 μL) of the supernatant was injected into the HPLC-ECD. 

### 2.5. Behavioral Test

#### 2.5.1. Open Field Test

The open field experiment is an efficient assay for evaluating the overall expression of motor deficits in mouse models of PD. In this experiment, the open field consisted of a plaza box (40 × 40 cm), and a fence (35 cm tall). Mice were placed individually in the middle of the box allowed to adapt to the new environment a few minutes. Then, their behavior was recorded on video for approximately 10 min. The box was cleaned with 70% alcohol and dried between each experiment to remove odor trails. Recording and analysis of behavior was performed using EthoVision Version XT 13 (Noldus Information Technology, Wageningen, The Netherlands).

#### 2.5.2. Rotarod Test

The rotarod test was conducted to evaluate rodent motor coordination. The rod hung 30 cm above a table covered with a mat with two gaskets on either side of the rod to anchor it. The method is modified from a previous study [26]. The rotation rod rotates at a linear speed from 0 rpm to 40 rpm in 300 s. When the animal falls off the rod, the automatic sensor captures and automatically calculates the total distance of movement (m). Before administration of the test, all animals were trained on the rotarod (5 rpm) facing away from the inspector. On the day of the test, the animals were positioned on the rotarod to assess their motor coordination expression, which was estimated by the ability of mice to stay on accelerating rotating rods. The fall delay was measured in the eight groups, with an interval of 10 min. The test cut-off time was 300 s.

### 2.6. Estimation of SOD, CAT and GPx Activity

First, the blood samples were centrifuged at 16,000× *g* for 10 min at 4 °C. The plasma was removed, and the red blood cells were gently mixed with triple-deionized water (1:4, v/v). Second, the samples were centrifuged at 16,000× *g* for 10 min at 4 °C. The supernatant was quantified using GPx, CAT and SOD assay kits, according to the manufacturers’ instructions.

Evaluation of SOD activity was performed using superoxide dismutase assay kit (Cayman, MI, USA). The kit was used to measure all three types of SOD. These SOD have been characterized according to their Fe, Mn and Cu/Zn contents. The kit uses a tetrazolium salt for the determination of superoxide radicals generated by xanthine oxidase. One unit of SOD was defined as the amount of enzyme needed to dismutate 50% of the superoxide radicals. Use a Tecan Infinite 200 Microplate Reader (Männedorf, Switzerland), the absorbance was read at 450 nm. [27].

The methods described in previous studies were modified to evaluate CAT activity [28]. A catalase assay kit (Cayman, MI, USA) was used to evaluate CAT activity through the decomposition of hydrogen peroxide. Using a Tecan Infinite 200 Microplate Reader (Männedorf, Switzerland), the absorbance was read at 540 nm.

A GPx assay kit (Cayman, MI, USA) was used to determine GPx activity. The method converts reduced glutathione into oxidized glutathione by GPx and oxidizes NADPH into NADP^+^. Using a Tecan Infinite 200 Microplate Reader (Männedorf, Switzerland), the absorbance was read at 340 nm. [29].

### 2.7. Immunohistochemistry

Urethane (1 g/kg i.p.) was used to anesthetize mice, and 0.9% normal saline was used to clean the blood remaining on the brain. The striatum tissues were soaked in 4% paraformaldehyde for a few hours, then transferred to 0.1 M phosphate-buffered saline (PBS) overnight, after which they were dehydrated and embedded in paraffin. The slices (5 µm thick) were fixed on silane-coated slides, deparaffinized using xylene twice, and then rehydrated sequentially in 100, 75, and 50% anhydrous alcohol. Antigen retrieval was performed through heating in sodium citrate buffer with Tween 20 at pH 6.0 for 20–40 min. The sections were incubated with 3% H_2_O_2_ for 20–30 min at ambient temperature to deplete endogenous peroxidase. After washing with PBS, the sections were incubated with anti-tyrosine hydroxylase Ab (Sigma T8700) at 1:1000 overnight at 4 °C. After rinsing with PBS, the sections were incubated with polyclonal goat anti-rabbit immunoglobulins/HRP (Dako P0448) 1:100 for 1 h and washed in PBS with Triton X-100 for 10 min. Then, the sections were visualized with diaminobenzidine (DAB), and the color change to brown, which occurred within 3–5 min, was monitored. The sections were dyed with hematoxylin, rinsed with deionized water, dried and sealed with sealing solution. Finally, the Aperio Scan Scope CS system (Leica, Wechsler, Germany) was used to capture images of the stained slices. Measurement of the number of TH-positive cells was performed using image analysis software (Aperio Image Scope, version, 12.3.3). TH-positive cells were evaluated according to the following formula:

IHC Index = (n1/T) × 1 + (n2/T) × 2 + (n3/T) × 3(1)

where n is the number of stained pixels per spot, and the subscripts 1, 2, 3 indicate weak, moderate, and strong staining, respectively. T is the total number of pixels per spot.

### 2.8. Method Validation for the Neurochemicals

Method validation was performed based on the US FDA guidelines for bioanalytical method validation (Guidance for Industry: Bioanalytical Method Validation, 2001), which contains methods for the lower limit of quantification, accuracy and precision, and the calibration curve. Linearity was determined in the range of 5–200 ng/mL. The correlation coefficient (r^2^) value of all calibration curves was greater than 0.995. The accuracy (bias %) was calculated from the mean value of the observed concentration (C_obs_) and nominal concentration (C_nom_) using the relationship accuracy (bias %) = [(C_obs_−C_nom_)/C_nom_] × 100. The relative standard deviation (RSD) was calculated from the observed concentrations as precision (RSD %) = [standard deviation (SD)/Cobs] × 100. Intraday and interday variations of the method in rat samples were less than ±15% (±20% at the lower limit of detection) for all analytes. The limit of quantification (LOQ) was defined as the lowest concentration of the linear range, and the limit of detection (LOD) was defined as the concentration of analyte with a signal-to-noise ratio (S/N) of 3.

### 2.9. Statistical Analysis

The data are presented as the mean ± SD. The graphs of all the experimental data were prepared using Sigma plot (version 10) software. Dunnett′s test was used to calculate statistical differences over two groups using ANOVA. The data were further analyzed by Student′s *t*-test. *p* < 0.05 indicates a significant difference.

## 3. Results

### 3.1. Analytical Method Validation

**Figure 2:**
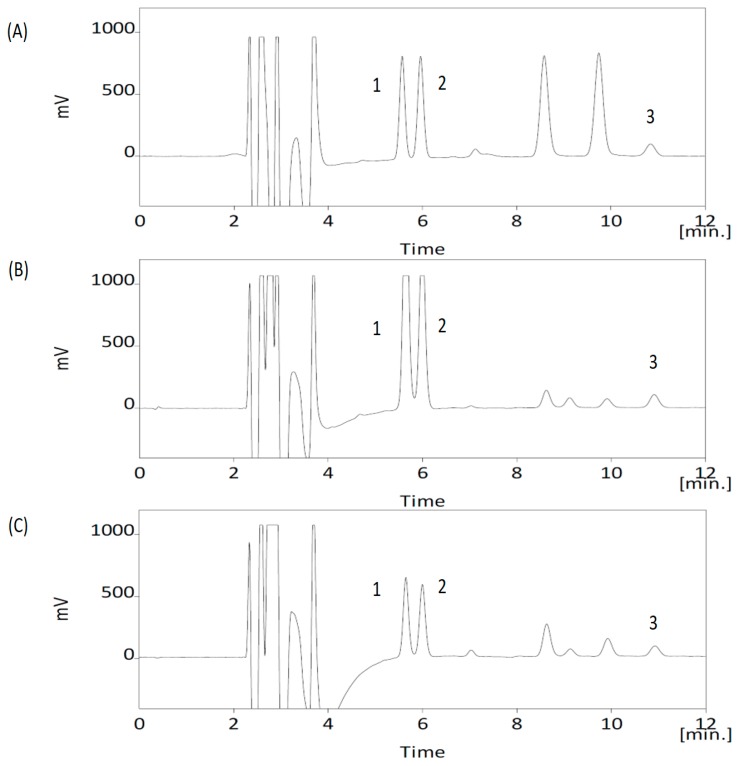
The HPLC-ECD chromatographs of neurochemicals (**A**) standard solution (100 ng/mL), (**B**) basal level striatum sample: (1) dopamine (471.14 ng/mL), (2) DOPAC (2013.33 ng/mL), (3) HVA (657.50 ng/mL), (**C**) MPTP group at striatum sample: (1) Dopamine (86.15 ng/mL), (2) DOPAC (104.93 ng/mL), (3) HVA (67.46 ng/mL). ECD, electrochemical detection.

The HPLC-ECD detection method was used to separate DA, DOPAC and HVA from mouse striatum samples. The representative chromatograms of striatum are shown in Figure 2. There was no interference under the analytical conditions at the retention time of DA, DOPAC and HVA, which were eluted at 5.6, 5.9 and 10.9 min, respectively. The linearity of each calibration curve, ranging from 5 to 200 ng/mL, was verified, and the coefficient of determination (r^2^) was greater than 0.995. The precision and accuracy in the striatum are presented in Table 1. Precision: RSD (%), relative standard deviation = [standard deviation/C_obs_] × 100; accuracy: bias (%) = [(C_obs_−C_nom_)/C_nom_] × 100. The accuracy and precision values within ±15% and were acceptable. The LOD and LOQ were determined at S/N (signal-to-noise) ratios of 3 and 10 under the present chromatographic conditions. The lower LOQ of lamivudine was determined to be 5 ng/mL.

### 3.2. Behavioral Experiments

#### 3.2.1. Rotarod Test

**Figure 3:**
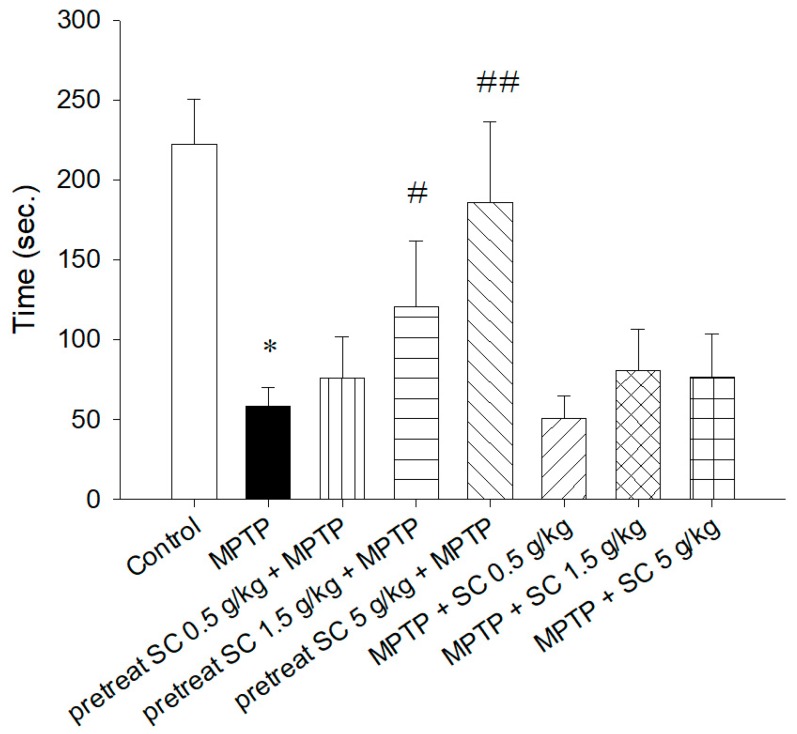
Rotarod performance in different experimental groups. The data are expressed as the mean ± SD. * *p* < 0.001 compared with the control group; # *p* < 0.005 and ## *p* < 0.001 compared with the MPTP group as determined by *T*-tests. MPTP, 1-methyl-4-phenyl-1,2,3,6-tetrahydropyridine; SC: *Schisandra chinensis* extract.

To assess the MPTP-induced impairment of motor coordination, all animals were positioned on a rotarod apparatus accelerating from 0 to 40 rpm to determine their retention time on the rotating rod after MPTP treatment. The data are presented in Figure 3. Compared with the control mice on day 4, the groups treated with MPTP alone showed a significant reduction in retention time (*p* < 0.001). In the pretreated groups, the mice that received medium (1.5 g/kg, p.o.) (*p* < 0.005) and high (5 g/kg, p.o.) (*p* < 0.001) doses of *S. chinensis* exhibited significantly improved rotarod performance compared with the group treated with MPTP alone. No significant difference in retention time was observed between the treated groups and the MPTP-only group. These results indicated that *S. chinensis* had a better prevention effect than a curative effect for MPTP-induced motor deficits.

#### 3.2.2. Open Field Test

**Figure 4:**
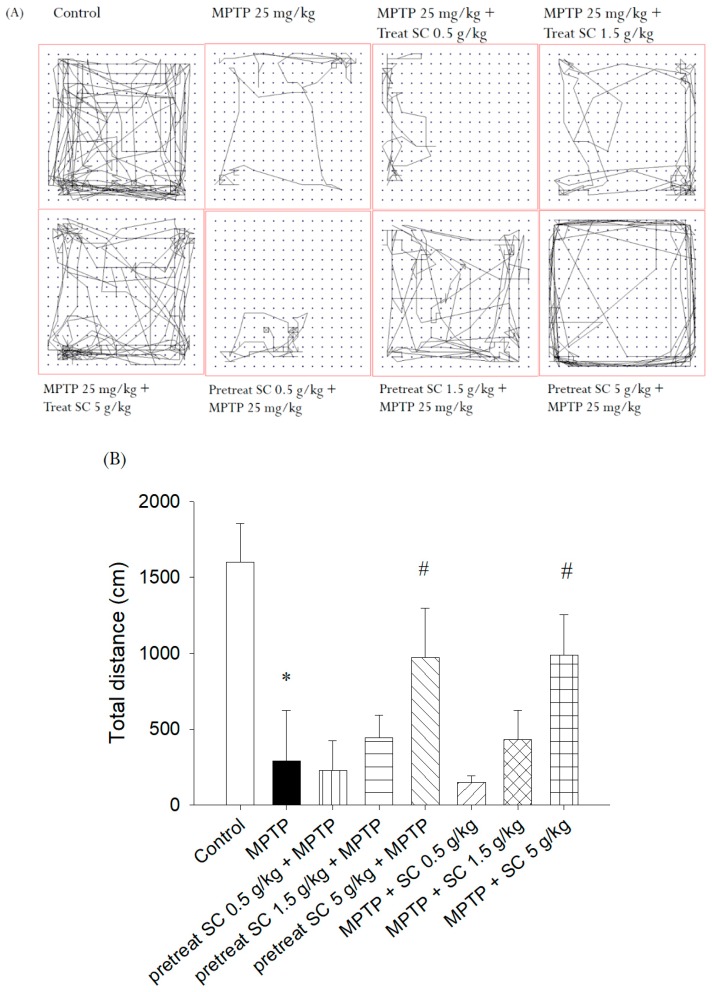
Effect of *S. chinensis* on total distance traveled as determined by movement traces in the open field test. (**A**) Mice exploring behavior in the open field test; (**B**) total distance within the experimental time. The movement distance of the MPTP-only group was significantly reduced compared with that of the control group; however, this reduction in motor activity was attenuated by pretreatment and treatment with high-dose *S. chinensis* (5 g/kg) compared with the MPTP-only group. The data are expressed as the mean ± S.D. (*n* = 6). * *p* < 0.001 compared with the control group; # *p* < 0.005 compared with MPTP group as determined by *t*-tests. MPTP, 1-methyl-4-phenyl-1,2,3,6-tetrahydropyridine; SC: *Schisandra chinensis* extract.

The open field experiment was used to assess locomotor, exploratory, and anxiety-like behaviors. A high frequency of these behaviors indicates increased locomotion and exploration and/or a lower level of anxiety. Figure 4 shows the effect of pretreatment and treatment after MPTP administration in mice on their locomotor activity in the open field. Figure 4A shows that MPTP-treated mice exhibited a significant reduction in open field activity compared with the control group of mice. Compared with the MPTP treatment alone (*p* < 0.005), the high dose (5 g/kg, p.o.) of *S. chinensis* in both the pretreated and treated groups significantly improved locomotor activity. The statistical analysis (Figure 4B) showed that MPTP lead to a decrease in the distance moved, but the high dose of *S. chinensis* in both the pretreatment and treatment groups improved these locomotor dysfunctions. The results of the open field experiment indicated that *S. chinensis* could ameliorate the behavioral impairments caused by MPTP intoxication and normalize the abnormal behavior of MPTP-treated mice.

### 3.3. Levels of Dopamine and Dopamine Metabolites in the Striatum

**Figure 5:**
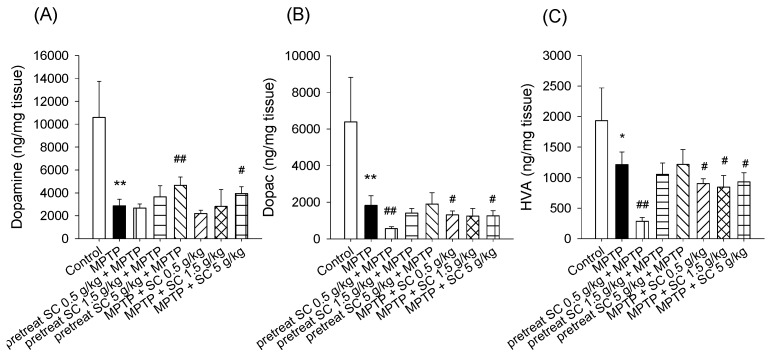
Effect of *S. chinensis* on the MPTP-induced loss of striatal dopamine (**A**) and its metabolites, (**B**) DOPAC and (**C**) HVA. The data are expressed as the mean ± S.D. (*n* = 6). * *p* < 0.05 and ** *p* < 0.001 compared with the control group using a t test; # *p* < 0.05 and ## *p* < 0.001 compared with the MPTP group as determined by *T*-tests. MPTP, 1-methyl-4-phenyl-1,2,3,6-tetrahydropyridine; SC: *Schisandra chinensis* extract.

To evaluate the amount of dopamine and its metabolites in the striatum, liquid chromatography coupled to electrochemical detection was performed. The results demonstrated that the levels of dopamine and its metabolites DOPAC and HVA were significantly decreased in the pretreated group and in the group treated with MPTP alone, which represented the successful establishment of the MPTP-induced PD experimental model compared with the controls (*p* < 0.001) (Figure 5) [30]. Compared with MPTP alone, the dopamine content was significantly increased in the high-dose *S. chinensis* (5 g/kg) pretreatment and treatment groups (*p* < 0.001, *p* < 0.05) (Figure 5). The effect on DOPAC and HVA level in the striatum region of all the experimental mice is depicted in Figure 5B,C. There was no significant change in the levels of DOPAC and HVA in the pretreatment groups of *S. chinensis* (1.5 and 5 g/kg).

### 3.4. Effect on SOD, CAT and GSH

**Figure 6:**
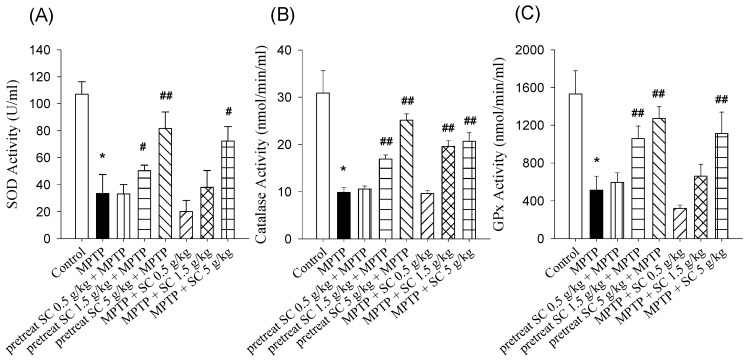
Effects of *S. chinensis* on the activity levels of SOD, CAT, and GPx in the blood of control and experimental mice. The levels of (**A**) SOD, (**B**) CAT and (**C**) GPx were significantly reduced in the MPTP-only group compared with that in the control group. Pretreatment and treatment with *S. chinensis* significantly increased the activities of SOD, CAT and GPx in the MPTP + SC group compared with the MPTP-only group. The data are expressed as the mean ± S.D. (*n* = 6). * *p* < 0.001 compared with control group; # *p* < 0.01 and ## *p* < 0.001 compared with the MPTP group as determined by *T*-tests. SOD, superoxide dismutase; CAT, catalase; GPx, glutathione peroxidase; MPTP, 1-methyl-4-phenyl-1,2,3,6-tetrahydropyridine; SC, *Schisandra chinensis* extract.

Oxidative stress plays a pivotal role in the neurodegeneration associated with PD; therefore, the impact of *S. chinensis* on the endogenous GPx and CAT and the antioxidant enzyme SOD was evaluated in the MPTP-induced experimental model (Figure 6). The levels of SOD, GPX and CAT were significantly reduced in MPTP-treated mice compared with the control group. Among the pretreatment groups, *S. chinensis* administration (1.5 and 5 g/kg, p.o.) significantly enhanced SOD activity in a dose-dependent manner (*p* < 0.01, *p* < 0.001). Among the treatment groups, a high dose of *S. chinensis* (5 g/kg, p.o.) significantly increased SOD activity compared with MPTP alone (*p* < 0.01) (Figure 6A). Both pretreatment and treatment with the high dose of *S. chinensis* alleviated the MPTP-induced impairment in SOD activity in the experimental animal model. Compared with the MPTP-only group, CAT activity was significantly increased following *S. chinensis* pretreatment (1.5 and 5 g/kg, p.o.) and treatment (1.5 and 5 g/kg, p.o.) (*p* < 0.001, *p* < 0.001) (Figure 6B). With regard to GPx, the groups pretreated with *S. chinensis* (1.5 and 5 g/kg, p.o.) and the group treated with *S. chinensis* (5 g/kg, p.o.) showed major recovery in GPx levels compared with the group that received MPTP alone (*p* < 0.001) (Figure 6C). 

### 3.5. Evaluation of Immunohistochemical Staining for Tyrosine Hydroxylase

**Figure 7:**
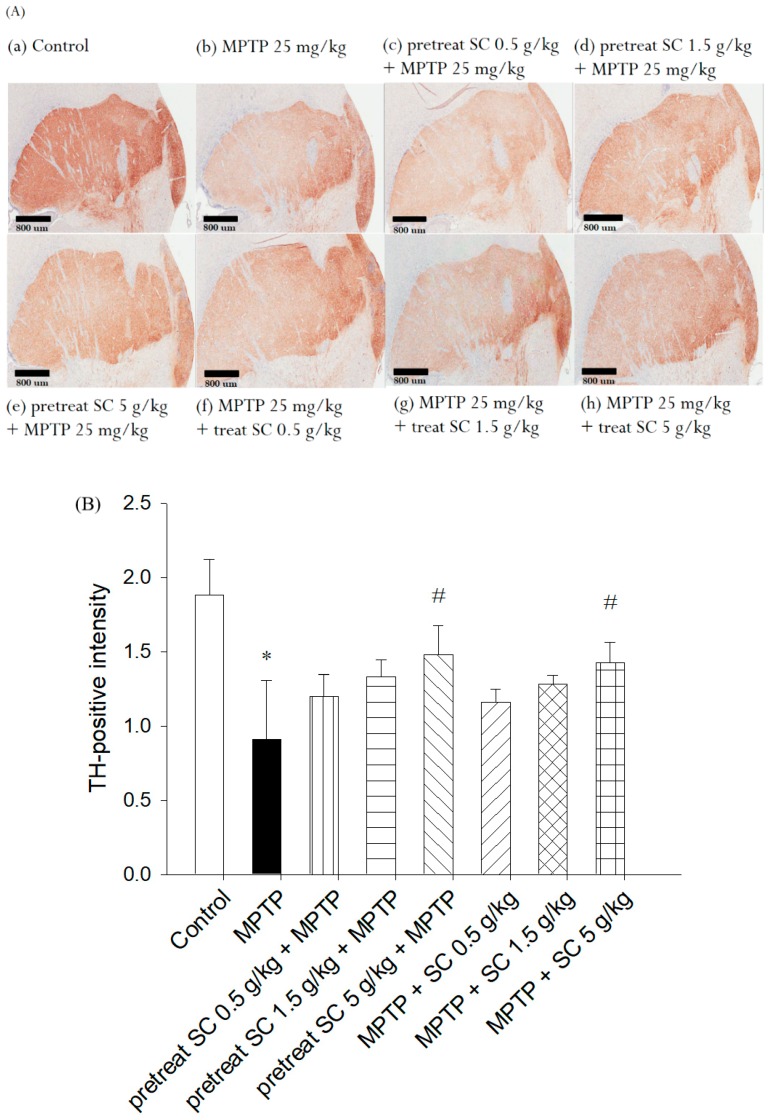
The effects of *S. chinensis* treatment (0.5, 1.5 and 5 g/kg, p.o.) on tyrosine hydroxylase (TH) in the striatum of control and experimental mice. (**A**) Immunohistochemical staining of TH (magnification, × 80) in the (a) control group; (b) MPTP group; (c) pretreatment *S. chinensis* (0.5 g/kg, p.o.) + MPTP group; (d) pretreatment *S. chinensis* (1.5 g/kg, p.o.) + MPTP group; (e) pretreatment *S. chinensis* (5 g/kg, p.o.) + MPTP group; (f) MPTP + *S. chinensis* (0.5 g/kg, p.o.) group; (g) MPTP + *S. chinensis* (1.5 g/kg, p.o.) group; and (h) MPTP + *S. chinensis* (5 g/kg, p.o.) group. (**B**) Quantitative results of TH staining by scoring the intensity of TH-positive cells in the striatum. The data are presented as the mean ± S.D. (*n* = 3). * *p* < 0.001 compared with the control group as determined by *T*-tests; # *p* < 0.05 compared with the MPTP (25 mg/kg, i.p.) group as determined by *T*-tests. MPTP, 1-methyl-4-phenyl-1,2,3,6-tetrahydropyridine; SC, *Schisandra chinensis* extract; TH, tyrosine hydroxylase.

To assess the functional viability of dopaminergic neurons in the striatum, the expression of the rate-limiting tyrosine enzyme, which is responsible for dopamine biosynthesis, was assessed using an anti-TH antibody. The results demonstrated that the control group exhibited a high number of TH-positive neurons (Figure 7A (a)). However, the level of TH was significantly decreased in the MPTP-treated (25 mg/kg, i.p.) group (Figure 7A (b)). Representative microphotographs of TH immunostaining in the SNpc demonstrated neuroprotective effect of *S. chinensis* (Figure 7A). Statistical results indicate that MPTP caused substantial neuronal loss in the SNpc. In contrast, *S. chinensis* at 5 g/kg significantly rescued the MPTP-induced loss of TH-positive neurons (Figure 7B). The results suggested that *S. chinensis* blocked MPTP-induced neurotoxicity of SNpc dopaminergic neurons in mice.

The statistical results demonstrated that, compared with the control group, the MPTP treatment (25 mg, i.p.) resulted in the loss of approximately 52% of neurons in the striatum. However, the groups pretreated and treated with *S. chinensis* (0.5, 1.5 and 5 g/kg, p.o.) improved and recovered the MPTP-induced loss of TH neurons (Figure 7B). Furthermore, a significant difference from MPTP treatment alone was observed with the high-dose pretreatment and treatment. These results suggest that high doses of *S. chinensis* may prevent and protect the MPTP-induced neurotoxicity of dopaminergic neurons in the mouse striatum. These results are in accordance with the behavioral experiments (Figure 3 and Figure 4), examination of neuronal dopamine level (Figure 5A), and evaluation of the endogenous antioxidant enzymes CAT, SOD and GPx (Figure 6).

## 4. Discussion

In our present work, we assessed the neuroprotective effect of *S. chinensis* extract in an animal PD model. The results indicated that *S. chinensis* extract protected against MPTP-induced locomotor dysfunction, dopaminergic neurons damage and oxidative stress in mice. The MPTP-treated experimental mice exhibited symptoms of PD, such as incoordination, tremor and bradykinesia. Locomotor dysfunction is considered a useful indicator of severity in animal models of PD. The rotarod and open field experiments were applied in our research. Open field was used to evaluate neurological deficits in mice. The *S. chinensis* extract significantly ameliorated MPTP-induced effects in mice with regard to mobility, including range of activity and movement distance. The reduction of TH-positive neurons reflects the severity of PD disease in the MPTP-induced group, and the immunohistochemistry results were in accordance with the behavior experiments. The results of the neurochemical experiments also indicated that *S. chinensis* blocked the MPTP-induced loss of striatum DA and its metabolism. Many studies have demonstrated that motor dysfunction in MPTP-induced mice can be observed through behavioral testing, including open field and rotarod experiments [31]. Our experimental data demonstrated that the groups pretreated and treated with *S. chinensis* showed extended time on the rotarod test compared with the MPTP-induced mice. This finding suggests a potential prevention and recovery effect of *S. chinensis* on the brain injury, which is consistent with the previous reports examining treatments with apigenin and luteolin [32,33].

Locomotor dysfunction, such as muscular rigidity, bradykinesia and tremor, is a clinical symptom of PD [34]. In the behavioral analysis, mice in the pretreated and treatment groups showed better muscle coordination and grip in the rotarod test and more locomotor activity in the open field test than the MPTP group. Both pretreatment and treatment with *S. chinensis* may protect dopaminergic cells from degeneration in the mouse striatum, leading to enhanced locomotion, motor coordination and overall behavioral activities. In particular, during the pretreatment stage, high doses of *S. chinensis* increased locomotor activity in the rotarod and open field tests, which correlated with increased physical performance and recovery rates [35,36].

After optimizing the analytical conditions, the following experiments were conducted to optimize the chromatographic separation of the analytes: chromatographic conditions, especially analytical columns and mobile phase compositions (concentration of buffer solution, pH value of the buffer solution and percentage of the organic solvent), were optimized to achieve good sensitivity and peak shape, as well as a relatively short run. It was observed that acetonitrile gave a better peak shape than methanol, and it was therefore selected as the organic phase. Finally, a mobile phase consisting of acetonitrile-buffer solution was used in the experiment. The representative chromatograms of each analyte under the optimized conditions are shown in Figure 2.

To evaluate the direct effect of *S. chinensis* on dopamine levels induced by MPTP, dopamine and its metabolites were quantified in the striatum. Our results demonstrated that the level of DA, DOPAC and HVA were significantly reduced in MPTP-induced mice, which is consistent with previous reports of PD patients [37]. Neurochemical data indicated that high-dose *S. chinensis* potentially blocked MPTP-induced loss of striatum dopamine in pretreated and treated groups. *S. chinensis* can increase dopamine levels, which may be because *S. chinensis* is rich in phytochemicals of lignans, vitamins and phenolic compounds. Previous studies have demonstrated that lignans and phenolic can protect neurons from toxicity induced by hydrogen peroxide and exhibit protective effects against neuronal cell death and neurodegenerative diseases [22,38]. The results indicate that *S. chinensis* provided the antioxidant and neuroprotective effect against the damage caused by MPTP. However, a low dose of *S. chinensis* extract in the pretreatment group and the full dose in the treated group resulted in decreased DOPAC and HVA levels, which may be because metabolic processes are generally complex, and DA levels may not be wholly consistent with their metabolites [39].

Impaired mitochondrial function and oxidative stress are two important factors caused by PD syndrome [40,41]. Many studies have demonstrated that injury to dopaminergic neurons during PD progression is associated with an increase in ROS [42]. According to previous studies, MPTP administration promotes ROS production and leads to dopaminergic neuronal damage [43]. In our study, we observed that MPTP administration also caused a significant increase in ROS levels, which was consistent with previous reports [43]. SOD, CAT and GPx effectively remove oxygen free radicals, which may be used to measure the level of oxidative stress. It has been reported that antioxidant treatment can partially protect animals from the neurotoxic effects of MPTPs [44]. In our study, the activity levels of SOD, CAT and GPx were significantly decreased in the MPTP-only group, suggesting that oxidative stress is involved in the pathogenesis of PD. Furthermore, pretreatment with *S. chinensis* (1.5 and 5 g/kg, p.o.) or treatment with *S. chinensis* (5 g/kg, p.o.) in MPTP-treated mice significantly improved the activity levels of GPx, CAT and SOD. These results demonstrated that *S. chinensis* potentially protects dopaminergic neurons by enhancing the antioxidant efficiency. This antioxidant capacity of *S. chinensis* can be attributed to its botanical ingredients, such as lignans, volatile oils, vitamins and organic acids [45]. The components of *S. chinensis* have been shown to exhibit neuroprotection in a model of neurodegeneration disease [46].

TH is a rate-limiting enzyme that limits the conversion of L-DOPA to dopamine [47]. The number of dopaminergic neurons and fibers in substantia nigra can be evaluated by TH immunohistochemistry [48]. In our study, TH immunohistochemistry showed that the dopaminergic neurons in striatum of MPTP-treated mice were significantly reduced. According to previous studies, the major cause of dopaminergic neuron loss is oxidative stress due to MPTP exposure [49]. The effect of *S. chinensis* in the pretreatment and treatment groups (5 g/kg) confirmed the neuroprotective effect. This protection may be related to the antioxidant and neuroprotective effects of *S. chinensis*.

## 5. Conclusions

The overall study described the neuroprotective effects of *S. chinensis* against MPTP-induced neurotoxicity in mice. PD is a major factor in oxidative stress, and here, it was demonstrated that if oxidative stress were alleviated, then PD symptoms will also be improved. Our study demonstrated that ethanolic-water extract of *S. chinensis* has a strong antioxidant property, which helped to prevent MPTP-induced neurotoxicity in mice. The *S. chinensis* extract improved behavioral disorders, oxidative stress-associated enzyme activities and dopamine levels and improved TH expression in the brain and protected the dopaminergic neurons. Furthermore, the neuroprotective effects are probably related to antioxidant mechanisms. Therefore, *S. chinensis* extract should be a potential candidate for nutritional supplement in the prevention or treatment of PD.

## Figures and Tables

**Table 1:** Method validation for the intra-assay precision (% RSD) and accuracy (% Bias) of the HPLC method for the determination of DA, DOPAC and HVA in the striatum.

Intraday				Interday		
Nominal Concentration (ng/mL)	Observed Concentration (ng/mL)	Precision (% RSD)	Accuracy (% Bias)	Observed Concentration (ng/mL)	Precision (% RSD)	Accuracy (% Bias)
DA						
5	4.70 ± 0.05	1.04	−5.99	4.32 ± 0.09	2.01	−13.68
10	10.16 ± 0.09	0.86	1.62	9.79 ±0.09	0.87	−2.11
50	50.18 ± 1.14	2.27	0.37	49.50 ± 1.16	2.34	−0.99
100	101.14 ± 1.87	1.85	1.14	103.43 ± 0.84	0.81	3.43
200	199.34 ± 1.15	0.58	−0.33	198.49 ± 2.45	1.23	−0.75
DOPAC						
5	4.33 ± 0.08	1.77	−13.43	4.31 ±0.01	2.28	−13.90
10	9.98 ± 0.05	0.54	−0.16	9.96 ± 0.08	0.79	−0.36
50	49.99 ± 0.24	0.48	−0.03	49.97 ± 0.29	0.58	−0.07
100	100.45 ± 0.29	0.29	0.45	100.47 ±0.31	0.30	0.47
200	199.31 ± 0.38	0.19	−0.34	199.10 ± 0.67	0.34	−0.45
HVA						
5	4.32 ± 0.08	1.94	−13.55	4.13 ± 0.12	2.82	−17.47
10	10.17 ± 0.20	1.99	1.74	10.31 ± 0.11	1.10	3.11
50	50.35 ± 0.22	0.43	0.70	50.81 ± 0.32	0.62	1.62
100	99.75 ± 0.06	0.06	−0.25	99.26 ± 0.21	0.21	−0.74
200	199.99 ± 0.06	0.03	−0.01	200.08 ± 0.12	0.06	0.04

The data are expressed as the mean ± S.D. (*n* = 5). DA: dopamine, DOPAC: 3-methoxy-4-hydroxyphenylacetic acid, HVA: homovanillic acid, RSD: the relative standard deviation.

## References

[1] Dehay B., Bourdenx M., Gorry P., Przedborski S., Vila M., Hunot S., Singleton A., Olanow C.W., Merchant K.M., Bezard E. (2015). Targeting α-synuclein for treatment of Parkinson’s disease: Mechanistic and therapeutic considerations. Lancet Neurol..

[2] Dauer W., Przedborski S. (2003). Parkinson’s disease mechanisms and models. Neuron.

[3] Klegeris A., McGeer P.L. (2000). R-(−)-Deprenyl inhibits monocytic THP-1 cell neurotoxicity independently of monoamine oxidase inhibition. Exp. Neurol..

[4] Zhang J., Perry G., Smith M.A., Robertson D., Olson S.J., Graham D.G., Montine T.J. (1999). Parkinson’s Disease Is Associated with Oxidative Damage to Cytoplasmic DNA and RNA in Substantia Nigra Neurons. Am. J. Pathol..

[5] Hartmann A. (2004). Hartmann A Postmortem studies in Parkinson’s disease. Dialogues Clin. Neurosci..

[6] Burns R.S., Chiueh C.C., Markey S.P., Ebert M.H., Jacobowitz D.M., Kopin I.J. (1983). A primate model of Parkinsonism Selective destruction of dopaminergic neurons in the pars compacta of the substantia nigra by MPTP. Proc. Natl. Acad. Sci. USA.

[7] Davis G.C., Williams A.C., Markey S.P., Ebert M.H., Caine E.D., Reichert C.M., Kopin I.J. (1979). Chronic parkinsonism secondary to intravenous injection of meperidine analogues. Psychiatry Res..

[8] Heikkila R.E., Hess A., Duvoisin R.C. (1984). Dopaminergic neurotoxicity of 1-methyl-4-phenyl-1, 2, 5, 6-tetrahydropyridine in mice. Science.

[9] Speciale S.G. (2002). MPTP: Insights into parkinsonian neurodegeneration. Neurotoxicol. Teratol..

[10] Nagatsu T., Sawada M. (2006). Molecular mechanism of the relation of monoamine oxidase B and its inhibitors to Parkinson’s disease: Possible implications of glial cells. J. Neural Transm..

[11] Javitch J.A., D’Amato R.J., Strittmatter S.M., Snyder S.H. (1985). Parkinsonism-inducing neurotoxin, MPTP uptake of the metabolite MPP+ by dopamine neurons explains selective toxicity. Proc. Natl. Acad. Sci. USA.

[12] Lee D.H., Kim C.S., Lee Y.J. (2011). Astaxanthin protects against MPTP/MPP+-induced mitochondrial Dysfunction and ROS production in vivo and in vitro. Food Chem. Toxicol..

[13] Dal Ben M., Bongiovanni R., Tuniz S., Fioriti E., Tiribelli C., Moretti R., Gazzin S. (2019). Earliest Mechanisms of Dopaminergic Neurons Sufferance in a Novel Slow Progressing Ex Vivo Model of Parkinson Disease in Rat Organotypic Cultures of Substantia Nigra. Int. J. Mol. Sci..

[14] Zhang C., Zhao X., Mao X., Liu A., Liu Z., Li X., Bi K., Jia Y. (2014). Pharmacological evaluation of sedative and hypnotic effects of schizandrin through the modification of pentobarbital-induced sleep behaviors in mice. Eur. J. Pharmacol..

[15] Zhu H., Zhang L., Wang G., He Z., Zhao Y., Xu Y., Gao Y., Zhang L. (2016). Sedative and hypnotic effects of supercritical carbon dioxide fluid extraction from Schisandra chinensis in mice. J. Food Drug Anal..

[16] Lee K., Ahn J.H., Lee K.T., Jang D.S., Choi J.H. (2018). Deoxyschizandrin, Isolated from Schisandra Berries, Induces Cell Cycle Arrest in Ovarian Cancer Cells and Inhibits the Protumoural Activation of Tumour-Associated Macrophages. Nutrients.

[17] Jiang Y.M., Wang Y., Tan H.S., Yu T., Fan X.M., Chen P., Zeng H., Huang M., Bi H.C. (2016). Schisandrol B protects against acetaminophen-induced acute hepatotoxicity in mice via activation of the NRF2/ARE signaling pathway. Acta Pharmacol. Sin..

[18] Yang G.Y., Wang R.R., Mu H.X., Li Y.K., Xiao W.L., Yang L.M., Pu J.X., Zheng Y.T., Sun H.D. (2011). Neolignans from Schisandra wilsoniana and Their Anti-human Immunodeficiency Virus-1 Activities. Chem. Pharm. Bull. (Tokyo).

[19] Xu L., Grandi N., Del Vecchio C., Mandas D., Corona A., Piano D., Esposito F., Parolin C., Tramontano E. (2015). From the traditional Chinese medicine plant Schisandra chinensis new scaffolds effective on HIV-1 reverse transcriptase resistant to non-nucleoside inhibitors. J. Microbiol..

[20] Huang H., Shen Z., Geng Q., Wu Z., Shi P., Miao X. (2017). Protective effect of Schisandra chinensis bee pollen extract on liver and kidney injury induced by cisplatin in rats. Biomed. Pharmacother..

[21] Song Q.Y., Gao K., Nan Z.B. (2016). Highly oxygenated triterpenoids from the roots of Schisandra chinensis and their anti-inflammatory activities. J. Asian Nat. Prod. Res..

[22] Lee T.H., Jung C.H., Lee D.H. (2012). Neuroprotective effects of Schisandrin B against transient focal cerebral ischemia in Sprague-Dawley rats. Food Chem. Toxicol..

[23] Wang M., Bi W., Fan K., Li T., Yan T., Xiao F., He B., Bi K., Jia Y. (2018). Ameliorating effect of Alpinia oxyphylla-Schisandra chinensis herb pair on cognitive impairment in a mouse model of Alzheimer’s disease. Biomed. Pharmacother..

[24] Yang B.Y., Han W., Han H., Liu Y., Guan W., Li X.M., Kuang H.X. (2018). Effects of Lignans from Schisandra chinensis Rattan Stems against Abeta1-42-Induced Memory Impairment in Rats and Neurotoxicity in Primary Neuronal Cells. Molecules.

[25] Reagan-Shaw S., Nihal M., Ahmad N. (2008). Dose translation from animal to human studies revisited. FASEB J..

[26] Rozas G., López-Martín E., Guerra M.J., Labandeira-García J.L. (1998). The overall rod performance test in the MPTP-treated-mouse model of Parkinsonism. J. Neurosci. Methods.

[27] Nandi A., Chatterjee I.B. (1988). Assay of superoxide dismutase activity in animal tissues. J. Biosci..

[28] Sinha A.K. (1972). Calorimetric assay of catalase. Anal. Biochem..

[29] Rotruck J.T., Pope A.L., Ganther H.E., Swanson A.B., Hafeman D.G., Hoekstra W.G. (1973). Selenium biochemical role as a component of glutathione peroxidase. Science.

[30] Sridharan S., Mohankumar K., Jeepipalli S.P., Sankaramourthy D., Ronsard L., Subramanian K., Thamilarasan M., Raja K., Chandra V.K., Sadras S.R. (2015). Neuroprotective effect of Valeriana wallichii rhizome extract against the neurotoxin MPTP in C57BL/6 mice. Neurotoxicology.

[31] Shi X., Chen Y.H., Liu H., Qu H.D. (2016). Therapeutic effects of paeonol on methyl-4-phenyl-1, 2, 3, 6-tetrahydropyridine/probenecid-induced Parkinson’s disease in mice. Mol. Med. Rep..

[32] Patil S.P., Jain P.D., Sancheti J.S., Ghumatkar P.J., Tambe R., Sathaye S. (2014). Neuroprotective and neurotrophic effects of Apigenin and Luteolin in MPTP induced parkinsonism in mice. Neuropharmacology.

[33] Rai S.N., Yadav S.K., Singh D., Singh S.P. (2016). Ursolic acid attenuates oxidative stress in nigrostriatal tissue and improves neurobehavioral activity in MPTP-induced Parkinsonian mouse model. J. Chem. Neuroanat..

[34] Jankovic J. (2008). Parkinson’s disease: Clinical features and diagnosis. J. Neurol. Neurosurg. Psychiatry.

[35] Ahumada F., Hola R., Wikman G. (1991). Effect of schisandra chinensis extract on thoroughbreds in sprine race. Equine Athl..

[36] Hancke J., Burgos R., Wikman G., Ewertz E., Ahumada F. (1994). Schisandra chinensis, a potential phytodrug for recovery of sport horses. Fitoterapia.

[37] Piggott M.A., Marshall E.F., Thomas N., Lloyd S., Court J.A., Jaros E., Burn D., Johnson M., Perry R.H., McKeith I.G. (1999). Striatal dopaminergic markers in dementia with Lewy bodies, Alzheimer’s and PD rostrocaudal distribution. Brain.

[38] Gu B.H., Minh N.V., Lee S.H., Lim S.W., Lee Y.M., Lee K.S., Kim D.K. (2010). Deoxyschisandrin inhibits H2O2-induced apoptotic cell death in intestinal epithelial cells through nuclear factor-ΚB. Int. J. Mol. Med..

[39] Laatikainen L.M., Sharp T., Harrison P.J., Tunbridge E.M. (2013). Sexually dimorphic effects of catechol-O-methyltransferase (COMT) inhibition on dopamine metabolism in multiple brain regions. PLoS ONE.

[40] Di Napoli M., Shah I., Stewart D. (2007). Molecular pathways and genetic aspects of Parkinson’s disease from bench to bedside. Expert Rev. Neurother..

[41] Schapira A.H. (2008). Mitochondria in the aetiology and pathogenesis of Parkinson’s disease. Lancet Neurol..

[42] Barnham K.J., Masters C.L., Bush A.I. (2004). Neurodegenerative diseases and oxidative stress. Nat. Rev. Drug Discov..

[43] Sriram K., Pai K.S., Boyd M.R., Ravindranath V. (1997). Evidence for generation of oxidative stress in brain by MPTP in vitro and in vivo studies in mice. Brain Res..

[44] More S.V., Kumar H., Kang S.M., Song S.Y., Lee K., Choi D.K. (2013). Advances in neuroprotective ingredients of medicinal herbs by using cellular and animal models of Parkinson’s disease. Evid. Based Complement. Alternat. Med..

[45] Panossian A., Wikman G. (2008). Pharmacology of Schisandra chinensis Bail: An overview of Russian research and uses in medicine. J. Ethnopharmacol..

[46] Sowndhararajan K., Deepa P., Kim M., Park S.J., Kim S. (2018). An overview of neuroprotective and cognitive enhancement properties of lignans from Schisandra chinensis. Biomed. Pharmacother..

[47] Lüdecke B., Knappskog P.M., Clayton P.T., Surtees R.A., Clelland J.D., Heales S.J., Brand M.P., Bartholomé K., Flatmark T. (1996). Recessively inherited L-DOPA-responsive parkinsonism in infancy caused by a point mutation in the TH gene. Hum. Mol. Genet..

[48] Fukuda T., Takahashi J., Tanaka J. (1999). TH-immunoreactive neurons are decreased in number in the cerebral cortex of PD. Neuropathology.

[49] Ghosh A., Roy A., Matras J., Brahmachari S., Gendelman H.E., Pahan K. (2009). Simvastatin inhibits the activation of p21ras and prevents the loss of dopaminergic neurons in a mouse model of Parkinson’s disease. J. Neurosci..

